# Enhancing sepsis biomarker development: key considerations from public and private perspectives

**DOI:** 10.1186/s13054-024-05032-9

**Published:** 2024-07-13

**Authors:** Jean-Francois Llitjos, Enitan D. Carrol, Marcin F. Osuchowski, Marc Bonneville, Brendon P. Scicluna, Didier Payen, Adrienne G. Randolph, Stephan Witte, Jesus Rodriguez-Manzano, Bruno François, on behalf of the Sepsis biomarker workshop group

**Affiliations:** 1 Open Innovation and Partnerships (OI&P), bioMérieux S.A., Marcy l’Etoile, France; 2 Anesthesiology and Critical Care Medicine, Hospices Civils de Lyon, Edouard Herriot Hospital, Lyon, France; 3 Department of Clinical Infection, Microbiology and Immunology, University of Liverpool Institute of Infection Veterinary and Ecological Sciences, Liverpool, UK; 4 Department of Paediatric Infectious Diseases and Immunology, Alder Hey Children’s NHS Foundation Trust, Liverpool, UK; 5 Ludwig Boltzmann Institute for Traumatology, The Research Center in Cooperation with AUVA, Vienna, Austria; 6 Medical and Scientific Affairs, Institut Mérieux, Lyon, France; 7 Department of Applied Biomedical Science, Faculty of Health Sciences, Mater Dei Hospital, University of Malta, Msida, Malta; 8 Centre for Molecular Medicine and Biobanking, University of Malta, Msida, Malta; 9 Paris 7 University Denis Diderot, Paris Sorbonne, Cité, France; 10 Departments of Anaesthesia and Pediatrics, Harvard Medical School, Boston, MA USA; 11 Department of Anesthesiology, Critical Care and Pain Medicine, Boston Children’s Hospital, Boston, MA USA; 12 AdrenoMed AG, Hennigsdorf, Germany; 13 Department of Infectious Disease, Faculty of Medicine, Imperial College London, London, UK; 14 Medical-Surgical Intensive Care Unit, Réanimation Polyvalente, Dupuytren University Hospital, CHU de Limoges, 2 Avenue Martin Luther King, 87042 Limoges Cedex, France; 15 Inserm CIC 1435, Dupuytren University Hospital, Limoges, France; 16 Inserm UMR 1092, Medicine Faculty, University of Limoges, Limoges, France

**Keywords:** Sepsis, Biomarker, Intensive care, Workshop

## Abstract

Implementation of biomarkers in sepsis and septic shock in emergency situations, remains highly challenging. This viewpoint arose from a public–private 3-day workshop aiming to facilitate the transition of sepsis biomarkers into clinical practice. The authors consist of international academic researchers and clinician-scientists and industry experts who gathered (i) to identify current obstacles impeding biomarker research in sepsis, (ii) to outline the important milestones of the critical path of biomarker development and (iii) to discuss novel avenues in biomarker discovery and implementation. To define more appropriately the potential place of biomarkers in sepsis, a better understanding of sepsis pathophysiology is mandatory, in particular the sepsis patient’s trajectory from the early inflammatory onset to the late persisting immunosuppression phase. This time-varying host response urges to develop time-resolved test to characterize persistence of immunological dysfunctions. Furthermore, age-related difference has to be considered between adult and paediatric septic patients. In this context, numerous barriers to biomarker adoption in practice, such as lack of consensus about diagnostic performances, the absence of strict recommendations for sepsis biomarker development, cost and resources implications, methodological validation challenges or limited awareness and education have been identified. Biomarker-guided interventions for sepsis to identify patients that would benefit more from therapy, such as sTREM-1-guided Nangibotide treatment or Adrenomedullin-guided Enibarcimab treatment, appear promising but require further evaluation. Artificial intelligence also has great potential in the sepsis biomarker discovery field through capability to analyse high volume complex data and identify complex multiparametric patient endotypes or trajectories. To conclude, biomarker development in sepsis requires (i) a comprehensive and multidisciplinary approach employing the most advanced analytical tools, (ii) the creation of a platform that collaboratively merges scientific and commercial needs and (iii) the support of an expedited regulatory approval process.

**Graphical Abstract:**

Conceptional approach to sepsis biomarker development.ED: emergency department; ICU: intensive care unit; PICU: paediatric intensive care unit
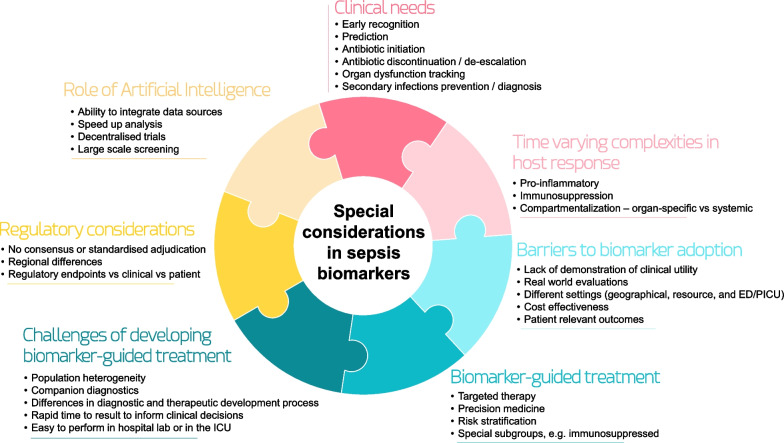

## Background

A biomarker is described as “a defined characteristic that is measured as an indicator of normal biological processes, pathogenic processes, or responses to an exposure or intervention, including therapeutic interventions”. This definition is supported by the FDA and the NIH and is used in the Biomarkers, Endpoints, and other Tools (BEST) [1]. This encompasses molecular, histologic, radiographic, or physiologic characteristics.

The development of biomarkers in the life-threatening context of sepsis is made difficult by several constraints. First, the development of a clinically useful sepsis biomarker requires multiple steps beyond finding an association between a particular molecule and a clinical state or outcome. Second, no clear roadmap exists for establishing how a biomarker should be approved for use in critically ill patients. Third, the definition of sepsis as a dysregulated host response to infection encompasses multiple heterogeneous subgroups in both adult and paediatric populations, therefore consistent biomarker-outcome relationships must be established while addressing the case-mix of septic patients. Further challenges in biomarker discovery include that more than one biomarker for pathways recognized to play a major role in sepsis pathophysiology is likely to be discovered, and the timing of intervention and targeted subgroup most likely to benefit must be defined for each. Interdisciplinary collaboration is crucial for biomarker adoption. It facilitates the integration of diverse expertise, fostering a comprehensive understanding of biomarkers’ multifaceted nature. However, it also presents a significant barrier due to the complexity of coordinating efforts across different disciplines. The lack of a common language and understanding can hinder effective communication and consensus-building, which could hamper successful validation and implementation of biomarkers in clinical practice.

To address this and foster interdisciplinary exchanges, the Institut Merieux organized a three-day meeting held November 27–29, 2023 and titled: “*An unmet sepsis challenge: facilitating the biomarker transition to clinical practice*” in Annecy, France. It involved stakeholders from the private and public sectors working on sepsis biomarkers research and innovation. The present review addresses many of the topics discussed and biomarker development strategies proposed during this meeting, which could help realising the full potential of sepsis biomarkers in improving patient care and outcomes.”. It discusses the stepwise development of sepsis biomarkers in the context of commercial development and marketing. Such a developmental process requires multiple interconnected steps beyond establishment of an association between a particular molecule and a clinical state and/or outcome. Finally, we propose a set of available solutions supported by several short-/long-term goals. A coordinated implementation of the above elements outlines a framework for streamlining biomarker research and application.

## The challenge: What is the place for biomarkers in sepsis?

This difficult question highlights both the current status of existing biomarkers and future biomarkers emerging from basic and translational research. Identification of future biomarkers will be facilitated by the rapid proliferation of cellular and molecular knowledge on the pathophysiology of sepsis facilitated by advances in "omics" technologies. In the last decade, several concepts have gained attraction in sepsis research: the essential role of the host response in sepsis severity and outcome [2]; the dynamic nature of the host immune response with oscillating and crossing waves of pro- and anti-inflammatory biomarker profiles [3, 4]; and the constant balance of the host response between resistance and tolerance. As genes underlying host susceptibility for sepsis are identified in broad genome studies [5, 6], the functionality of the genes (transcriptomic) and their protein expression (proteomic) is now being interrogated [7–11]. Notably, gene expression candidate biomarkers were shown to outperform traditional, advocated protein biomarkers, particularly distinguishing between differing infectious aetiologies [12, 13], and COVID-19 [14]. A more recent investigation has also highlighted the potential diagnostic value of integrating host transcriptomics and plasma metagenomics for sepsis diagnosis [15]. Single cell transcriptomics is also emerging as a more holistic tool to discovering sepsis biomarkers by defining functional cellular states [8, 16]. Moreover, it is essential to integrate the influence of environmental conditions (i.e. exposome), the presence of chronic inflammation and ageing, and the respective development and senescence of the immune system in young children and the elderly, respectively [17, 18]

Sepsis clinical characterization and outcome prediction need more than clinical parameters and scores across heterogeneous populations. The global failure of trials testing one molecule interfering with different host response pathways, suggests that underlying mechanisms for sepsis are complex and must be better elucidated. Interest is growing for studying sepsis subtypes (aka sub-phenotypes, subgroups), to characterize the human host response, with the aim of identifying targeted therapies for use in patients who will benefit [19, 20]. The demonstrated time variations of host response to infection also imposes more than biomarker measurement upon admission. Host response may be amenable to therapeutic modulation according to the immune profile-related timing, virulence of pathogens and clinical subtypes.

## Clinical needs around host biomarkers in sepsis

Sepsis is a catchment term for all patients with a systemic dysregulated host response to severe infection, and therefore is heterogeneous in relation with predisposition, type of pathogens, and pre-sepsis conditions. The clinical trajectory of these patients and their outcomes is also highly variable. Despite this, numerous studies have shown that early recognition and intervention with fluids, vasoactive agents and antimicrobials decreases sepsis morbidity and mortality [21–23]. Unfortunately, therapeutic management of sepsis remains limited outside of supportive care for varying levels of organ dysfunction. Many biomarkers have been associated with sepsis severity and outcomes, but their clinical utility for optimizing use of effective targeted therapies remains to be proven. A detailed roadmap for determining when a sepsis biomarker is ready for clinical use is needed for moving the field forward, and it must address the challenges outlined above (Table 1).

**Table 1:** Key Future Directions and Needs in Sepsis Biomarker Research

Direction	Justification
Multi-Marker/Source Approach	A panel of biomarkers that reflect different aspects of the immune response, organ dysfunction, and microbial presence provides a more comprehensive understanding of sepsis phenotype and improves diagnostic accuracy
Dynamic Monitoring	Sepsis rapidly progresses through distinct phases with varying disease manifestations. Biomarkers that capture these dynamical changes will enable more specific interventions and assessment of treatment efficacy over time
Use of Artificial Intelligence	Analysis of Big Data originating from various biological sources and capturing various sepsis phenotypes provides multitude diagnostic and predictive permutations. Use of AI will enable selection of the most accurate/reliable biomarker algorithms
Validation and Reproducibility	Robust biomarker validation ensures their superior reliability and reproducibility across diverse patient populations. Independent studies based on multicentre collaborations will strengthen the utility of identified biomarkers
Collaboration and Data Sharing	Collaborative efforts among researchers, clinicians, and industry stakeholders enable the pooling of resources, expertise, and data. Open sharing of information accelerates biomarker development and validation, ultimately benefiting patient care
Unified Regulatory Assessment/Approval	Meeting regulatory standards is a critical step in biomarker development. A unified assessment and approval process of biomarkers by various national regulatory agencies will exaccelate clinical implementation while maintaining uniform safety, efficacy, and quality criteria

**Fig. 1:**
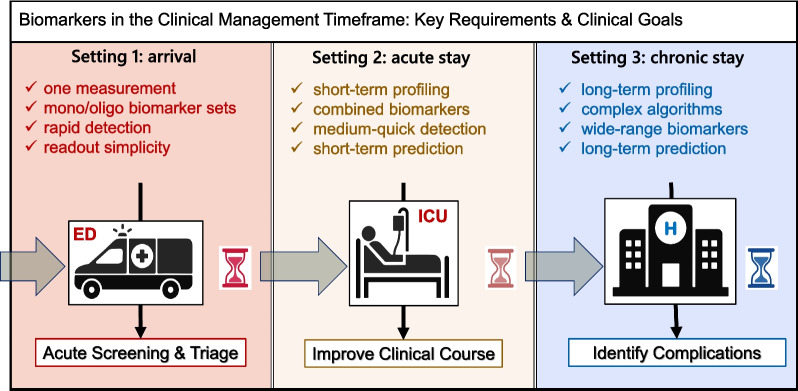
Biomarkers in the clinical management timeframe: key requirements & clinical goals. *ED* emergency department; *ICU* intensive care unit

In the context of developing laboratory biomarkers for sepsis based on the host response, the serial timeframe for clinical management can be broadly categorized into three settings: (i) arrival at the emergency department (ED), (ii) the early phase of the stay in intensive care unit (ICU), and (iii) during prolonged stay in ICU. Biomarker needs in these settings differ. In the ED, an objective of diagnostic biomarkers is to distinguish those patients without bacterial sepsis for whom it is safe to withhold antibiotics, and for prognostic biomarkers for triage of patients at risk of deteriorating and developing life-threatening organ dysfunction requiring escalation to a higher level of care. In the early phase of ICU management, biomarkers are often used to assess the trajectory of sepsis by tracking the severity of organ dysfunction. While organ dysfunction biomarkers are not per se indicators of sepsis, they must be an integral element of the patient monitoring profiles; sepsis affects parenchymal organs such as kidney, heart and liver [24] while the SOFA and Phoenix Sepsis Score-based readouts are at the centre of defining sepsis in both adult [25] and paediatric [26] populations. In both the ED and early phase of ICU management, a predictive biomarker could be used to identify patients that may benefit from a targeted treatment, facilitating rapid initiation with the aim of hastening recovery. Finally, during the prolonged stay in ICU, biomarkers may aid in prevention proposing to stimulate immunity or to early diagnose and treat secondary infections to minimize their harm (Fig. 1).

In the ED, rapid decision-making is crucial to assess ongoing infections and the risk of progression to life-threatening organ dysfunction and death. Differentiating between viral and bacterial infections is important to limit unnecessary antimicrobial treatments. Current tests such as procalcitonin (PCT), C-reactive protein (CRP) alone or combined with TRAIL and IP-10 [27] have limitations in accurately identifying bacterial or viral infections. Moreover, there are no approved tests for specific pathogen-associated biomarkers that could specify type/genre of the invading pathogen and optimize antibiotic treatment. There has been a robust growth of genomic/molecular methodologies that are capable of precise detection of bacterial RNA/DNA in the body fluids [28]. These approaches display high sensitivity, but their clinical utility is typically hindered by low/varying specificity. However, this field holds promise and e.g., recent SUSPECTS (suppression PCR-based selective enrichment sequencing) diagnostic platform was able to detect eight different (most common) sepsis-causative pathogens [29]. Research has yet to definitively exclude bacterial or viral infection within a timeframe to safely implement a therapeutic strategy. Therefore, a short time to result is an important prerequisite in this setting. It is also useful to consider endpoints that could help adapt and narrow treatment, especially using tests which are faster and more efficient than conventional microbiology to guide antibiotic de-escalation.

Due to early recognition of sepsis and improved management, the patients surviving the early phase of sepsis remain at risk of secondary ICU-acquired infections, which are associated with increased mortality, ICU readmissions, longer ICU stay, and long-term sequelae. These infections are linked with biological markers of inflammation, coagulopathy and endothelium damage (especially robust in COVID-19), metabolic and immune dysfunction [30–32]. Therefore, it is of pivotal interest to develop multiparametric biomarkers to identify patients at risk of developing ICU-acquired infections or more specific organ failures and to subgroup patients more likely to respond to treatments at different time points. The development of theragnostic biomarkers that provide relevant information on the underlying mechanism is strongly recommended.

### Age-related difference in sepsis must be considered

The bulk of the existing sepsis literature focuses on patients in their 50’s and 60’s. Sepsis studies in the very young and the very old are more limited but important to consider given the incidence of sepsis in early life, mostly in children under the age of five years [33]. Prematurity and low birth weight are major risk factors for poor sepsis outcomes in the neonatal period. The presence of co-morbidities is generally lower in children compared to adults however, survival rates have markedly increased for babies born prematurely, with genetic-metabolic disorders or with congenital malformations, and for children with oncologic, respiratory, cardiac, and neuromuscular disorders. These children now comprise a significant proportion of septic paediatric patients hospitalized for febrile illness and life-threatening infection [34, 35].

Primary immunodeficiencies, though rare, also emerge during childhood, sometimes presenting as severe sepsis [36].

Although sepsis is common in children, less than 1% of young patients evaluated for infection in the ED will develop life-threatening sepsis [37]. The immune system of children differs from adults, and evolves over time, influenced by exposure to pathogens and vaccines. The interferon response to pathogens becomes robust early in life, while the humoral immune response and the antigenic repertoire of T cells increase during childhood reaching adult levels during late adolescence [38]. Early-onset changes in inflammatory, endocrinologic, and metabolic pathways in septic patients are similar in children and adults [39], however, there is a wide variation in physiology between the neonatal to late adolescent phase of childhood. In part, this is because most children have healthy hearts that tolerate high levels of tachycardia, a nonspecific response to stress. This response enables children to initially maintain blood pressure in septic shock. However, once this compensatory response is overcome, infants and young children have lesser reserves to compensate for serious illness and may rapidly decline, thus shortening the window of opportunity for clinicians to recognize life-threatening infection. Therefore, application of biomarker-based risk-stratification is crucial to identify these cases for early triage and intervention before clinical deterioration.

Clinically, determining the origin of a fever is of paramount importance in paediatrics. The same questions and challenges as with adult sepsis arise: is it bacterial, viral, parasitic, or non-infectious inflammatory? What is the specific pathogen? Which patients are at risk for deterioration? Although there is an important overlap of treatment signatures between children and adults, cut-offs associated with specific diagnoses and outcomes likely differ. For instance, procalcitonin values are higher in the first 72 h of life than in childhood and adulthood [40–43]. Suppression of tumorigenicity 2 (ST2) protein concentrations increase in childhood, especially in males [44]. Pancreatic stone protein (PSP) concentrations vary across age, with lowest values in premature new-borns, followed by a rise through childhood to adolescence, and are lower in adults than children [45]. The above-mentioned separation is also justified by the so-called “inflammaging” frequently present in aged patients [17, 46]. A state of sterile, chronic, and low-grade inflammation combined with senescence of immune-inflammatory cells can markedly alter the diagnostic readouts. Multiplex host biomarker assays, including transcriptomic signatures, are being tested for use in distinguishing multiple diseases at once, including bacterial, viral, inflammatory, malaria, and immune status [https://www.diamonds2020.eu] but the age factor must be accounted for in those characteristics. However, the assessment of actual medical value impacting paediatric treatments and outcomes remains to be determined in randomised controlled trials.

The immune system experiences substantial modifications in the very elderly (> 80 years old). Immunosenescence commonly occurs, characterized by a decrease in immune cell counts or lymphopenia, and a diminished variety of variable receptor genes found on B and T cells with immunosuppressive cell proliferation together with release of anti-inflammatory cytokines [4, 17, 47]. Consequently, older persons are rendered more susceptible to acute viral and bacterial infections due to an insufficient immune response [48]. Approximately 65% of adults aged 65 to 84 years suffer of at least two comorbidities [49], which are typically linked to the several distinct pathophenotypes such as inflammation/immune response, thrombosis/haemorrhage, fibrosis, proliferation and apoptosis/necrosis. [50]. In contrast, healthy young adults and adolescents have a similar robust immune repertoire to fight infection [51]. Even in healthy young individuals, the immune response in sepsis is dysregulated and deleterious. A better understanding of these age-related differences across the lifespan is essential for developing sepsis biomarkers.

## Time-varying complexities of the host immune response in sepsis

The host immune response is precisely synchronized over time, with primary activation of sensory innate immune cells subsequent to pathogen recognition, followed by first order cytokine release, effector cell functions that include pathogen killing, and depending on the type of lymphocyte, activation of adaptive immune responses and resolution to homeostasis [52, 53]. Analyses of blood samples obtained from sepsis animals and patients have revealed the coexistence of two major arms of the host immune response, including inflammatory and immunosuppressive events [54–56]. An optimal immune response can be defined as a balance between efficient pathogen clearance and an acceptable level of immunopathology. Tolerance mechanisms limit the tissue damage induced by pathogens, and immunopathology [57] allows for the maintenance of a greater magnitude and duration of the immune response. Although some mechanisms regulating immunopathology tolerance and disease tolerance are functionally related, these two phenomena are clearly distinct. Tolerance refers to the mechanisms that render cells, tissues, organs, or organisms tolerant to deleterious stimulators that would otherwise be more destructive. This protection mainly depends on cellular metabolic reprogramming, which varies in time and space, using distinct gene expression programs and different metabolic programs. The processes for resistance (anabolic metabolism) require large amounts of energy characterized by an increase of the aerobic glycolysis (known as a Warburg effect) associated with inhibition of catabolism pathways of oxidative phosphorylation [58]. The balance between resistance (anabolic) and tolerance (catabolic) processes is controlled by the mammalian target of rapamycin (mTOR) [59]. Inflammatory signals inhibit the mTOR pathway and block oxidative phosphorylation by stimulating aerobic glycolysis. Tolerance mechanisms include diverse processes that act at the tissue and cellular levels, such as activation of the HPA axis, fatty acid oxidation, and IL-10 and IL-4 signalling [58].While an overwhelming inflammatory response is typically associated with increased acute mortality, sustained immune suppression is associated with the occurrence of secondary infections and late mortality [60]. It has been demonstrated that features of both phenomena (i.e. of hyperinflammation and immunosuppression) occur simultaneously in patients in sepsis [2, 20, 61], and their dynamic interplay defines an ultimate immune-inflammatory status of a given patient.

It is of paramount importance to develop time-resolved tests for diagnosing and assessing the persistence of these major immune phenomena to guide use of specific treatment strategies, including immunomodulatory drugs. To do so, several factors must be incorporated. First, we need to consider the existence of both quantitative and functional compartmentalization of the immune response, with significant variations over time and between organs [61]. Indeed, in a state of homeostasis, the number and nature of immune cells vary greatly from one organ to another [62–64]. Similarly, the functional response of immune cells to infection varies in intensity and nature from organ to organ. Second, most biological investigations are carried out on peripheral circulating blood due to its ease of accessibility, and the influence of this limitation should be considered in the development of biomarkers for diagnostic and/or risk stratification strategies.

In addition, biomarker research traditionally has concentrated on measuring single time points, predominantly on ICU admission in the context of sepsis. This approach runs the risk of overlooking important changes and patterns in the levels of biomarkers that could offer valuable insights into causality and response to treatment. Repeated measurements, which entail gathering data at several time intervals, provide a more thorough perspective on host responses. For example, changes in expression of key inflammatory proteins and genes S100A8 and S100A12 were indirectly correlated to those of CD74 and HLA-DR over the course of recovery from septic shock [65]. Serial monitoring of biomarkers can provide valuable insights into patient reactions to medications, and disease progression [66]. Moreover, serial measurements may provide opportunities to modify the duration of antibiotic therapy thereby safely limiting a patients’ exposure to broad-spectrum antibiotics. In the ongoing biomArker-guided Duration of Antibiotic treatment in hospitalized Patients with suspecTed Sepsis (ADAPT-Sepsis) trial [67], the aim is to determine if the duration of antibiotic treatment for sepsis patients can be safely decreased through the daily monitoring of PCT and CRP levels. Incorporating serial biomarker measurements requires the use of sophisticated statistical methods to handle the intricacies of longitudinal data. Analytical strategies should address the multi-collinearities in repeated measurements, address missing data appropriately, and include both within-subject and inter-subject variability in order to differentiate significant changes from random fluctuations. Statistical models that include mixed-effects models, time-series analysis, and machine learning algorithms can provide the needed framework to address these challenges.

## Identified barriers to biomarkers adoption in sepsis

Despite significant progress in sepsis biomarker research, several barriers hinder the widespread adoption of the existing markers into clinical practice, which span scientific, clinical, logistical, and regulatory aspects (Table 2). A major adoption barrier is the lack of evidence of the clinical utility of biomarkers in sepsis, for either diagnosis, stratification or prognosis. There is a need for a more concerted clinical validation through high-performance (e.g. adaptive design) randomized clinical trials to assess their utility. International multi-centre trials [68–70] rapidly set up during the COVID-19 pandemic are examples to follow. Furthermore, these studies should be performed in a real-world setting across different geographical/ethnic contexts, and need to be focused on specific patient cohorts with defined clinical syndromes rather than on entire populations covering highly heterogeneous sepsis patients. Incorporating cohorts established in lower and/or middle-income areas will significantly enhance our goals of resolving sepsis heterogeneity and establishing precision medicine in sepsis. A too simplistic biomarker evaluation across all septic patients with a view to demonstrate their broadest possible value may lead to repeated failures [71], thus hampering biomarker assessment in the most relevant patient subsets [72].

**Table 2:** The most relevant barriers in adoption of the available sepsis biomarkers

Heterogeneity of Sepsis Phenotypes
Lack of Consensus on Definitions
Limited Specificity/Sensitivity
Cost and Resources Implications
Methodologic Validation Challenges
Regulatory Hurdles
Integration with Clinical Decision-Making
Interdisciplinary Collaboration
Limited Awareness and Education

From an economic standpoint, the level of evidence of the cost effectiveness of using existing biomarkers is currently low and mainly restricted to the value of PCT to guide antimicrobial discontinuation [73, 74]. Robust health economics and outcome research could help determine how a given biomarker could bring in the most benefits through proper integration in existing diagnostic algorithms. Determinants of prescribing various biomarker-based tests and their effects on patient management and outcomes should also be considered. The evaluation must also identify factors limiting adoption of those biomarkers, such as impact on organizational changes induced by the change of routine practice.

Another key limiting factor is the currently used sepsis biomarker evaluation methodology. To date, there are no clear recommendations and/or guidelines defining the performance requirements for such tests apart from routine design and statistical requirements by the national regulatory agencies. Given the complexity of sepsis (i.e. a “sepsis syndrome” is an umbrella-descriptor embracing dozens of infection-driven phenotypes), the performance requirements for methodological evaluation of biomarkers in sepsis are likely to vary according to the outcome considered and the population assessed. For instance, ruling out sepsis is a relevant clinical question at early disease stage requiring a focus on the biomarker negative predictive value (NPV). Along the same line, one could consider not accounting for non-predictable complications (e.g. unexpected haemorrhagic shock following catheter insertion in a septic shock patient). Thus, creation of new blueprints tailored for methodologic evaluation of sepsis biomarkers is warranted to enhance efficacy of markers (Table 2).

Finally, a significant barrier to the effective use of biomarkers in sepsis clinical practice is the absence of universally accepted management guidelines and the lack of consensus. Whereas numerous international initiatives have been performed [75–77], several challenges remain. Primarily, the landscape of potential sepsis biomarkers is vast, each possessing unique advantages and limitations. The intricate task of determining which biomarkers to incorporate into guidelines, and their subsequent interpretation, necessitates comprehensive research and clinical validation. Secondly, the absence of international collaboration in this domain is evident. Diverse geographical regions employ distinct methodologies for sepsis management, often lacking synchrony, thereby complicating the formulation of universally applicable guidelines. Lastly, the scarcity of resources and funding for such initiatives is a significant barrier. The development and implementation of international guidelines is a resource-intensive process, and many institutions lack the requisite resources for its execution. This underscores the need for concerted efforts in resource mobilization and international collaboration to advance sepsis management.

## Biomarkers guiding clinical decision in other fields: lessons learned from oncology

Cancer and sepsis are heterogeneous pathologies that involve multiple and complex mechanisms, for which a “one size fits all” approach is not sufficient. The evolution of oncology treatments has paralleled the development of molecular biomarkers enabling patients to be stratified according to their oncogenic pathways. Mutations of human epidermal growth factor receptor 2 (HER2) in breast cancer, anaplastic lymphoma kinase (ALK) in non-small cell lung cancer and v-raf murine sarcoma viral oncogene homolog B1 (BRAF) in melanoma constitute successful examples of how distinct pathophysiological malignancy traits can be characterized. These and other molecular stratification markers have emerged as a pivotal strategy in guiding the choice of treatment [78, 79]. In oncology, several biomarkers, like genetic mutations or chromosome alterations associated with a particular oncogenic pathway, are binary, i.e. they allow clear distinction between patients carrying or not these alterations. This dichotomic feature rarely applies to sepsis circulating biomarkers.

Moreover, the time required to assess the presence or expression level of a given biomarker in oncology can be several days, which is unthinkable in the case of a serious infection requiring extremely rapid management in the initial sepsis phase. However, there is encouraging work in sepsis, comparable to the enrichment strategies of certain immune or targeted therapies in oncology, based on evaluation of biomarkers, for example sTREM-1, bio-adrenomedullin, or monocyte expression of HLA-DR associated with particular pathophysiological mechanisms occurring in some sepsis patients [80–82]. One area of development in oncology which could inspire approaches to sepsis is that of quantitative evaluation of mutations (tumour mutational burden), which correlate with efficacy of some immunotherapies or metabolic control of cellular growth. Beyond their combination and level of expression, it would be useful to define a set of biomarkers assessing pathways involved in sepsis, and whether their multimodal appraisal is of interest.

Management of cancer patients undergoing immunosuppressive therapy and at risk of developing a serious infection or cancer recidivism is another topic of shared interest. Early identification of immunosuppressed patients could improve their prognosis. In addition, since these patients are very often excluded from interventional studies in sepsis, setting up dedicated studies would improve the level of evidence for most current and planned sepsis therapies.

## The promise of diagnostic-guided interventions

In the last three decades, beside transient approval of activated protein C [83], almost all trials that have tested innovative sepsis drugs have failed despite numerous promising preclinical data and early phase results. More recently, specific attention has been given to biomarker-guided intervention to select more appropriately patients [84] with a rigorous standardized methodology [85]. The COVID-19 pandemic conditions offered a unique opportunity to rapidly obtain large cohorts with sequential blood samples for biomarker measurements to propose appropriate drugs [86]. The classification of the patient cohorts in the sub-phenotype for immune profile guided the use of immunomodulatory drugs providing “personalized immunotherapy” [87]. If most of the major trials during COVID-19 have missed the opportunity to sample blood for pragmatic reasons during a frenetic period, some bring important results to adequately guide the use of tocilizumab and anakinra. Following this COVID-19 experience, European groups have developed trials in sepsis using a similar strategy to test immunomodulatory drugs based on longitudinal measurements of biomarkers (GM-CSF, presepsin, HLA-DR, etc.).This type of biomarker-guided approach will open the door to personalized therapeutic strategies taking into consideration the pathological process, the disease stage and individual patient characteristics [88]. In addition, biomarkers can help predict response to treatment but can also be correlated with outcome which gives a strong push for predictive and prognostic enrichment [89]. These innovative approaches also raised the interest of developing new clinical endpoints besides all-cause mortality at Day-28 for biomarker-guided personalized trials.

Several candidate biomarkers have been investigated and advocated for sepsis diagnosis and clinical management. Classically, procalcitonin (PCT) and C-reactive protein (CRP) have been prominent targets as candidate sepsis biomarkers. CRP, a well-known and relatively inexpensive inflammatory biomarker, has been used consistently in the context of adult and neonatal sepsis diagnosis [90–92]. Elevated PCT levels were shown to be associated with bacterial sepsis, organ dysfunction, and mortality, suggesting a role as a diagnostic and/or prognostic indicator [93, 94]. Moreover, randomized clinical trials demonstrated the effectiveness of monitoring PCT levels in antimicrobial stewardship and associated complications [95]. Notably, PCT has been highlighted as potential standout biomarker for distinguishing between viral and bacterial sepsis [96]. However, PCT (and CRP) levels gathered upon admission may not be helpful in identifying bacterial co-infection among patients with COVID-19 pneumonia [97]. Presepsin appears promising in early-onset sepsis diagnosis in adult or neonatal sepsis, since detectable levels increase early during the host response to infection [98–102]. Circulating nucleosomes have been identified as potential predictive biomarkers for sepsis and sepsis-associated organ dysfunction, offering diagnostic and prognostic value [103]. Studies have also raised concerns regarding the specificity and sensitivity of candidate biomarkers. Of note, the overarching message is that candidate biomarkers may not perform reliably as stand-alone sepsis biomarkers. For example, the use of stand-alone CRP or PCT remains uncertain [93, 104]. However, in combination with other markers and routine clinical scores, candidate biomarkers may assist in the early prediction of sepsis, for example PCT in combination with qSOFA [105].

When looking into the sepsis drug development pipeline, two drugs to be described briefly below have already integrated these criteria and are currently under evaluation, aiming for regulatory approval: Nangibotide and Enibarcimab are two examples of biomarker-guided theragnostic treatments of septic shock in sepsis that could be the first novel therapies available soon for the treatment of patients.

First example is Nangibotide, a synthetic TREM-1 antagonistic peptide that inhibits the TREM-1 receptor [106]. In phase 2 trial, Nangibotide showed benefit in the high sTREM-1 group of patients with septic shock based on predefined SOFA endpoints [80]. Nangibotide currently enters clinical phase 3. Second example is Adrenomedullin; its high circulating concentration correlates with mortality in sepsis and septic shock [107]. Recently, the AdrenOSS-2 phase 2a trial guided by elevated Adrenomedullin suggested benefit in patients treated with adrenomedullin antibody, Enibarcimab (previously adrecizumab) [81]. Interestingly, when adding another biomarker (DPP3) to the Adrenomedullin, a significant mortality signal was detected [108]. Beyond these two examples of biomarker-guided specific therapies targeting specific pathophysiological pathways, biomarkers allowing assessment of the inflammatory/immune status of sepsis patients (like HLA-DR) could be enrichment strategies for any targeted or non-targeted interventions aiming at either boosting or suppressing immune responses. Biomarkers could also guide interventions to prevent or treat sepsis complications. In this regard, recent trials have shown that, in a selection of patients undergoing cardiac surgery, using blood biomarkers associated with high risk for acute kidney injury (AKI) (like TIMP-2) reduced the rate and severity of AKI [109, 110]. This warrants future clinical evaluation of biomarker-guided approaches to improve management of septic AKI, using TIMP-2 or septic AKI endotypes or sub-phenotypes [111].

Even if many drugs appear promising and could be available at the bedside in the coming years, as seen in cancer, it remains to be seen whether a treatment guided by a single biomarker or more complex biomarker sets is more advantageous in reducing sepsis morbidity and mortality.

## Challenges in developing diagnostic-guided treatments

Sepsis heterogeneity has been identified as one of the main drivers for past clinical trial failures [112]. Studies have typically applied an ensemble of approaches, for example, defining baseline ranges from diverse cohorts, statistical model adjustments that account for baseline characteristics, sophisticated normalization procedures that accommodate for confounders, fitting multivariate models and machine learning algorithms to detect complicated patterns [7, 113–117]. The use of biomarkers for the selection of the patient population most likely to benefit from the therapy is currently considered the most promising approach [118]. It is also of utmost importance to understand the molecular mechanisms that drive alterations in biomarkers by employing experimental models to validate their significance in relation to the disease condition [13, 20].

Diagnostic-guided treatment approaches require the parallel development of a diagnostic assay and a therapeutic agent, which are different in several aspects. The processes require largely different technological skills and scientific knowledge and importantly, distinct development and registration pathways need to be followed and aligned. In the EU, the two components are regulated by different bodies. Most innovative therapeutics are approved centrally for all European countries by the European Medicines Agency (EMA), while diagnostics are regulated in the decentralized manner by “Notified Bodies”.

Clinical trials requiring a diagnostic test for patient selection are also much more complex. Diagnostic tests and devices need to be developed prior to the first clinical trial. As sepsis patients need to be screened in the ICUs also during nights and weekends, the diagnostic test must be quick and user friendly in addition to fulfilling the mandatory technical and analytical validation criteria. At this development stage, point-of-care tests are probably desired, allowing for testing directly in the ICU.

When it comes to commercialization of the drug, biomarker testing in the hospital’s central lab is usually desired to facilitate seamless integration into the hospital's treatment procedures for sepsis. This requires transitioning of the testing method to other platforms to be compatible with established test systems.

Overall, the multifaceted nature of a combined development for a therapeutic along with a companion diagnostic demands a comprehensive and adaptive approach to overcome the obstacles of bringing personalized medicines in acute care especially for the treatment of septic shock to success.

## The regulatory standpoint

From a regulatory standpoint, there are no recommendations dedicated to the development of biomarkers in sepsis. Even though this allow creativity and encourages innovative approaches, it is also a barrier to research and development. The lack of consensus regarding the level of statistical performances, such as specificity or negative predictive value, to be achieved in this setting is a common topic of discussion. Indeed, the definition of optimal thresholds is frequently left to the discretion of experts and to the clinical situation. The absence of performance targets to be reached during the development phase of diagnostic strategies can prolong delays. However, iterative exchanges with regulatory agencies during the development process make it possible to identify the desired targets, but this requires the ability to mobilize important resources. Recommendations issued by the Foundation for Innovative Diagnostics (FIND) in 2016 for biomarkers aimed at distinguishing bacterial from viral infections, could be a good starting point for the development of such guidelines [119, 120]. Notably, there are important differences between the FDA and the EMA in terms of exchanges, guidance and complexity in regulatory processes. Any discussion on biomarker development should also consider ethical aspects, such as patient consent and data privacy, especially when dealing with genetic markers. Finally, standardized adjudication protocols endorsed by scientific societies could probably be useful. Indeed, setting up clinical validation studies is costly and time-consuming, and requires a clear and consistent vision of inclusion and exclusion criteria.

## Artificial intelligence as panacea?

The advent of artificial intelligence (AI) and machine learning (ML) algorithms has ushered in a new era in sepsis biomarker development, potentially offering unprecedented opportunities for innovation and discovery [121]. The integration of AI and ML into biomarker research holds immense promise, driven by their ability to combine biological, clinical, and digital data streams to predict patient outcomes with unparalleled accuracy and precision [122]. Central to the transformative potential of AI and ML is their ability to refine and prioritize candidate biomarkers for inclusion in predictive models. The critical task of feature selection for biomarker discovery is underscored by the increasing size of databases and electronic records, together with advancements in data acquisition and computational analysis. However, the exponential growth of data also amplifies the risk of overfitting models, wherein the model becomes too finely tuned to the training data, leading to diminished reproducibility and challenges in platform transferability.

To circumvent these challenges, it is important to limit the number of features in models and to identify the “minimal signature” [123]. With this aim, recent work suggests the value of large language models (LLM) to prioritize candidate genes for inclusion in models [124]. Ultimately this speeds up the time needed to analyse the characteristics of the features included on the basis of pre-existing data, without having to dispense with secondary human validation. Furthermore, the concept of federated learning emerges as a viable solution to address privacy concerns and facilitate continuous machine learning progress. By decentralizing model training and allowing data to remain localized, federated learning not only enhances privacy but also promotes collaborative advancements in AI-driven biomarker development. Another proposal would be to consider sequential approaches, characterized by large-scale screening followed by targeted analyses on a smaller subset of patients. This iterative methodology, ideally suited for immunological analyses, enables the identification of pertinent biomarkers for more specific tests among the vast landscape of data [125].

From a more technical standpoint, ensuring the successful translation of sepsis biomarkers from discovery to implementation platforms necessitates that ML algorithms effectively tackle the issue of performance loss. This discrepancy often arises due to discrepancies between the discovery environment and real-world implementation settings. Therefore, it is crucial to consider factors such as the detection chemistry of the implementation platform, as well as the genomic context of the identified biomarkers [126]. Tailoring preselected features to uncover signatures that align with the requirements of the end-point diagnostic test ensures optimal biomarker translation for practical application. By addressing these challenges, researchers can maximize the translational potential of AI-driven biomarker discovery.

In essence, while the emergence of AI heralds a new era of possibilities in biomarker discovery, its true potential lies in its ability to navigate the complexities of data integration, model optimization, and translational research. As researchers continue to leverage the power of AI in the pursuit of novel sepsis biomarkers, collaborative efforts and innovative methodologies will pave the way towards transformative breakthroughs in clinical practice. AI is not a panacea, but a tool to accelerate biomarker discovery. Errors in algorithms can result in harm and inefficiency, and implicit biases in data sets used to train algorithms can entrench and even amplify existing problems [127].

## Conclusions

Undoubtedly, the progress in sepsis biomarker research has been painstakingly slow and marred by countless failures. Yet, “*It does not matter how slowly you go so long as you do not stop*” (Confucius, 551–479 BC), thus, it is crucial that the research community should continue advancing sepsis biomarker development. In recent years, the field has undergone significant advancements, and several promising directions in biomarker research for sepsis are emerging.

While key focus of future biomarker research in sepsis appears to lie in the identification of novel biomarkers that can offer desired sensitivity and specificity, one should not dismiss a potential utility of the past “failed” biomarkers. A robust proliferation of molecular and cellular markers [128, 129] (e.g., microRNAs, cell-free DNA) should not eclipse the need for re-testing of the old biomarkers (e.g. IL-6, TNF). The latter must be performed in a coordinated manner, focusing on recognizing distinct clinical scenarios/endpoints/risks rather than a one-size-fits-all diagnostic approach.

Intuitively, the future of sepsis biomarker research involves the development of multi-marker panels that can provide a more comprehensive and nuanced understanding of the disease. Such routine multi-marker panels could mimic “liquid biopsies” successfully used in the cancer field [130]. Whenever feasible, such liquid biopsies should be supplemented by relatively simple tissue biopsies (e.g. muscle, fat, urine, regional venous blood vs systemic) and broncho-alveolar fluid (e.g. in pneumonias) to expand the desired diagnostic knowledge to other compartments beyond the peripheral blood. Sepsis is a complex syndrome with diverse clinical manifestations, and a single biomarker will never capture its full complexity but rather a defined but short-lived element of the sepsis pathophysiology. Various omics-based technologies provide additional analytical fuel for unravelling known and unknown but intricate molecular pathways underlying sepsis. Finally, there is an eminent place for AI-driven integration of those multiple biomarkers from liquid blood biopsies, tissues and routine clinical data. By rapidly defining various elements of sepsis pathophysiology based on large-data integrative algorithms, AI has a realistic potential to aid medical personnel towards enhanced diagnostic accuracy, prognostic precision and eventually individualizing the treatment course of the patients.

As technology and understanding of sepsis pathophysiology continue to evolve, these advancements in biomarker research are poised to revolutionize sepsis management, ultimately improving patient outcomes and reducing the burden of this life-threatening condition. Biomarker development in sepsis requires (i) a comprehensive and multidisciplinary approach employing the most advanced analytical tools, (ii) the creation of a platform that collaboratively merges scientific and commercial needs and (iii) the support of an expedited regulatory approval process. A merger of the above factors will collectively contribute to the translation of biomarker discoveries from the laboratory to impactful clinical applications, which will improve patient outcomes and quality of life.

## Data Availability

Data sharing is not applicable to this article as no datasets were generated for this review.

## Abbreviations

AI: Artificial Intelligence
CRP: C-Reactive Protein
ED: Emergency Department
EMA: European Medicines Agency
FDA: U.S. Food and Drug Administration
ICU: Intensive Care Unit
LLM: Large Language Model
NPV: Negative Predictive Value
PCT: Procalcitonin
SIRS: Systemic Inflammatory Response Syndrome
